# Evaluating assessment tools of the quality of clinical ethics consultations: a systematic scoping review from 1992 to 2019

**DOI:** 10.1186/s12910-020-00492-4

**Published:** 2020-07-01

**Authors:** Nicholas Yue Shuen Yoon, Yun Ting Ong, Hong Wei Yap, Kuang Teck Tay, Elijah Gin Lim, Clarissa Wei Shuen Cheong, Wei Qiang Lim, Annelissa Mien Chew Chin, Ying Pin Toh, Min Chiam, Stephen Mason, Lalit Kumar Radha Krishna

**Affiliations:** 1 Division of Supportive and Palliative Care, National Cancer Centre Singapore, Level 4, 11 Hospital Drive, Singapore, 169610 Singapore; 2 Yong Loo Lin School of Medicine, National University of Singapore, 1E Kent Ridge Road, NUHS Tower Block, Level 11, Singapore, 119228 Singapore; 3 Lee Kong Chian School of Medicine, Nanyang Technological University, 59 Nanyang Dr, Experimental Medicine Building, Singapore, 636921 Singapore; 4 Medical Library, National University of Singapore Libraries, National University of Singapore, Blk MD6, Centre, 14 Medical Dr, #05-01 for Translational Medicine, Singapore, 117599 Singapore; 5 Department of Family Medicine, National University Health System, 5 Lower Kent Ridge Road, Singapore, 119074 Singapore; 6 Division of Cancer Education, National Cancer Centre Singapore, Level 4, 11 Hospital Drive, Singapore, 169610 Singapore; 7 Palliative Care Institute Liverpool, Academic Palliative & End of Life Care Centre, University of Liverpool, Liverpool, UK; 8 Cancer Research Centre, University of Liverpool, 200 London Road, Liverpool, L3 9TA UK; 9 Centre of Biomedical Ethics, National University of Singapore, Blk MD11, 10 Medical Drive, #02-03, Singapore, 117597 Singapore; 10 Duke-NUS Medical School, 8 College Road, Singapore, 169857 Singapore

**Keywords:** Clinical ethics committees, CECs, Clinical ethics consultations, Medical ethics, Clinical ethics

## Abstract

**Background:**

Amidst expanding roles in education and policy making, questions have been raised about the ability of Clinical Ethics Committees (CEC) s to carry out effective ethics consultations (CECons). However recent reviews of CECs suggest that there is no uniformity to CECons and no effective means of assessing the quality of CECons. To address this gap a systematic scoping review of prevailing tools used to assess CECons was performed to foreground and guide the design of a tool to evaluate the quality of CECons.

**Methods:**

Guided by Levac et al’s (2010) methodological framework for conducting scoping reviews, the research team performed independent literature reviews of accounts of assessments of CECons published in six databases. The included articles were independently analyzed using content and thematic analysis to enhance the validity of the findings.

**Results:**

Nine thousand sixty-six abstracts were identified, 617 full-text articles were reviewed, 104 articles were analyzed and four themes were identified – the purpose of the CECons evaluation, the various domains assessed, the methods of assessment used and the long-term impact of these evaluations.

**Conclusion:**

This review found prevailing assessments of CECons to be piecemeal due to variable goals, contextual factors and practical limitations. The diversity in domains assessed and tools used foregrounds the lack of minimum standards upheld to ensure baseline efficacy.

To advance a contextually appropriate, culturally sensitive, program specific assessment tool to assess CECons, clear structural and competency guidelines must be established in the curation of CECons programs, to evaluate their true efficacy and maintain clinical, legal and ethical standards.

## Introduction

Facing shifts in sociocultural paradigms, resource pressures and increasing complexities of medical care [1], the role of Clinical Ethics Committees (CEC)s has evolved. Whilst retaining its original role in facilitating “*the process and outcomes of patient care by helping to identity, analyze, and resolve*” ethical, moral and legal issues in clinical care [2] CECs have come to adopt active roles in education and policy making. To meet these goals, the CEC which is understood to be *“[a team of] physicians, social workers, attorneys, and theologians…which serves to review the individual circumstances of ethical dilemma and which has [previously shown to provide] much in the way of assistance and safeguards for patients and their medical caretakers”* [3] now educate patients, their families, clinicians, and the host organization as it guides them through the conflicts and uncertainties impacting their specific healthcare situation [4, 5]. CECs have also engaged in policy making roles to ensure consistency, transparency and accountability in resolving ethical issues in the clinical setting [3, 4].

Acknowledging these wider roles that have fuelled the expansion of CECs in North America [6, 7], Asia [8–12] and Europe [13–15], the American Society for Bioethics and Humanities (ASBH) – a key educational organization focused on advancing clinical and academic bioethics in the United States – has proposed a list of Core Competencies for clinical ethics consultants [16]. It is held that meeting these core competencies would allow CEC members to meet their new roles and responsibilities as well as prevailing clinical, ethical, professional and legal standards of practice [17, 18]. The ASBH’s Core Competencies also help set out the compositions of the CECs [19–21], inform the structuring [22–24] and monitoring of the content [5], quality [17, 18] and accountability [5, 25, 26] of CEC consultations (henceforth CECons) [19, 20, 27] and offer a means of ensuring the long-term viability of CECs [17, 28, 29].

However despite the establishment of ASBH’s Core Competencies, there is little means of assessing the quality of CECons [19, 20, 27].

### Need for this review

Focusing upon determining if and how CECs meet their ‘fundamental’ role of carrying out CECons and if these consults meet prevailing requirements, a systematic scoping review (SSR) of prevailing tools to assess the quality of CECons is proposed. This narrow area of study sets this SSR apart from previous reviews of CECs that have taken a more generalized view of assessing CEC function [30, 31]. It is hoped that mapping prevailing methods of assessing CECons will guide the design of a robust CECons assessment tool. This need to address this lack of an assessment tool to evaluate the approach, quality and content of CECcons [32], assess its long-term effects on patient care and safety [6, 33] and standardise and benchmark practice [34] is further underlined by evidence of variations in CEC practice and CECcons methods that will ultimately undermine the efficacy and standing of CECs as a whole. Better understanding of how CECs meet this key role will also improve oversight and improvements to quality standards and guidelines of CECs [35, 36].

## Methods

An SSR of prevailing methods and tools to assess CECons is proposed to map the size and scope of available literature in peer-reviewed and grey literature studies [37–41]. The flexible nature of an SSR enables systematic extraction and synthesis of actionable and applicable information [42] across a wide range of practice settings [43, 44], whilst summarizing available literature on CECons assessments [45, 46] and circumnavigating limitations posed by a dearth of relevant literature [43, 44, 47–49]. This data along with the identification of commonalities within CEC practice could lay the foundations for a consistent approach to assessing CECons [37–41].

Levac et al’s (2010) [50] adaptation of Arksey and O’Malley’s (2005) [37] methodological framework for conducting scoping reviews was adopted to map “*the key concepts underpinning a research area and the main sources and types of evidence available”* [40] and to *“produce a profile of the existing literature in a topic area, creating a rich database of literature that can serve as a foundation”* to inform practice and guide further research [38, 51, 52]. Guided by PRISMA-P 2015 checklist [45], a six-stage systematic scoping review protocol was developed for this study [37–41].

### Stage 1: identifying the research question

To better understand prevailing CECons assessment tools, the ten-member research team discussed prevailing concerns regarding evaluations of CECons with a team of experts consisting of two medical librarians, five CEC members at the National Cancer Centre Singapore and Singapore General Hospital; academics from the Centre for BioMedical Ethics at the National University Singapore and the Palliative Care Institute Liverpool at the University of Liverpool; and clinicians and educationalists from the Yong Loo Lin School of Medicine at the National University of Singapore (NUS) and Duke-NUS Medical School (henceforth the expert team).

To further focus this review on assessments of CECons, Post et al. (2015)‘s description of CECons was adopted to guide this process – areas to consider included “*the goals of ethics consultation, who may perform ethics consultation, who may request ethics consultations, what requests are appropriate for the ethics consultation service, what requests are appropriate for ethics case consultation, which consultation model(s) may be used and when, who must be notified when an ethics case consultation has been requested, how the confidentiality of participants will be protected, how ethics consultations will be performed, how ethics consultations will be documented, who is accountable for the ethics consultation service and how the quality of ethics consultation will be assessed and assured”* ([4], p.144)*.* From this description, it is evident that assessments of the CECons must necessarily include evaluations of personnel and the processes involved in CECons, the methods used to assess CECons and the outcomes of the CECons.

To this end, the expert and research teams determined the primary research question to be “what tools are available to evaluate the quality of CECons?” The secondary research questions include “what domains of CECons were evaluated in prevailing assessment tools, or were proposed to be evaluated?” and “how were they assessed, or proposed to be assessed?”

These questions were designed on the population, concept and context elements of the inclusion and exclusion criteria [53], using a PICOS format (Table 1). The draft protocol was designed and shaped by feedback from the panel of experts and research team.

**Table 1:** PICOS

PICOS	Inclusion	Exclusion
Population	Stakeholders directly involved within the CECon process	Stakeholders not directly involved within the CECon process
Intervention	Formal assessments of prevailing CECon practice	Description of CECon activities without any relation to assessment in any way.
Comparison	Comparison of prevailing methods and domains of CECon assessment	
Outcome	Outcome measures and challenges faced in assessing CECon practices, curricula and programs	
Study Design	Articles formally assessing the quality of CECons as well as papers that discussed the assessment of the quality of CECons Published between 1st January 1992 and 17th December 2019	Non-English articles without English translations. Opinion articles were excluded.

### Stage 2: identifying relevant studies

Independent pilot searches were carried out by the ten members of the research team using variations of “clinical ethics consultations” and “assessment” that appeared in titles and abstracts of research papers in PubMed between 1st January 1992 and 17th December 2019. The searches were confined to articles published after 1992, in acknowledgment of the year the Joint Commission’s first recognized the CEC’s role in patient care [4]. The detailed search strategy for PubMed is shown in Table 1 in the Additional file 1. Based on these findings the research team guided by the expert team created the search terms and strategies for the other databases.

The research team adopted the search strategies set out for each database and carried out independent searches of each database. The results of the independent pilot searches were discussed online and at face-to-face meetings where Sambunjak et al. (2010)‘s ‘negotiated consensual validation’ approach was used to achieve consensus on the final list of abstracts to be included [54]. Guided by the expert team, the research team conducted independent searches of PubMed, Embase, JSTOR, ERIC, Scopus and PsycInfo databases between 18th October 2019 to 17th December 2019.

### Stage 3: selecting studies to be included

Each member of the research team independently screened the titles and abstracts using the same screening tool. The list of articles identified by each member of the research team were shared and discussed online and at face-to-face meetings. ‘Negotiated consensual validation’ approach was employed to achieve consensus on the final list of full text articles to be studied and analyzed.

### Stage 4: data characterisation and analysis

With a focus on evaluating personnel, process and engagement of stakeholders in CECons, three members of the research team adopted Hsieh and Shannon’s approach to directed content analysis (2006) to independently assess the included articles [55, 56]. Four categories were drawn from Adams et al’s (2014) [57] review of Single-IRBs, Chenneville’s IRB Researcher’s Assessment Tool [58] and the core characteristics of CECs highlighted by Post et al. (2015) [4] and Flamm (2012) [59].

Concurrently in keeping with Krishna’s ‘Split Approach’ [60] that was adopted to enhance the trustworthiness and reproducibility of the analysis, three other members of the study team employed Braun and Clarke’s (2006) [61] thematic analysis approach to independently analyze the included articles. All articles were analysed through independent use of thematic analysis and directed content analysis. Use of Krishna’s ‘Split Approach’ [60] served as a means of confirming and triangulating the findings [62]. Concurrently, ‘negotiated consensual validation’ served as a means of peer debrief thus enhancing their validity [54, 63].

### Stage 5: collating, summarizing, and reporting the results

**Fig. 1:**
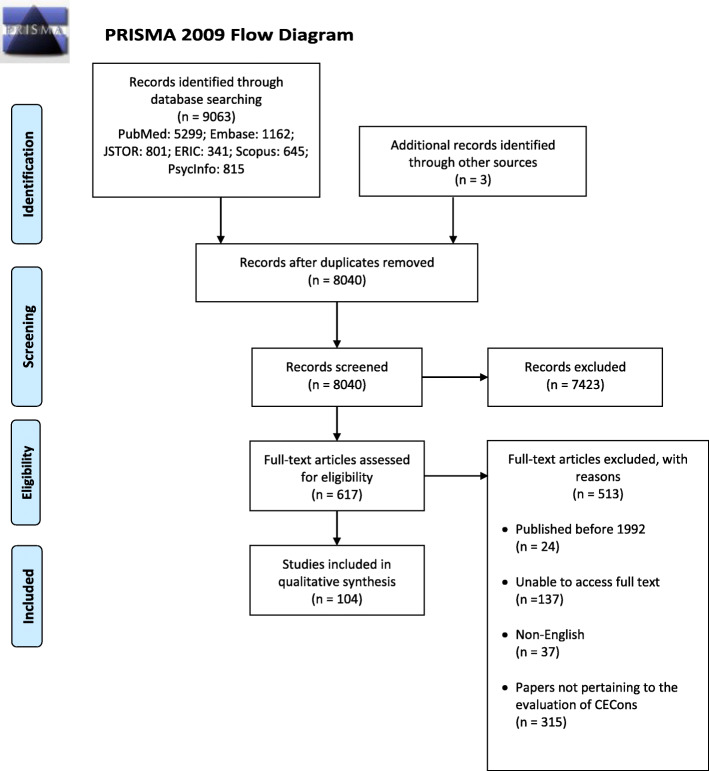
PRISMA Flowchart

Nine thousand sixty-six abstracts were identified, 617 full-text articles were reviewed and 104 full text articles were analyzed. (Fig. 1: *PRISMA Flowchart*). When compared, the findings of concurrent thematic and content analysis revealed the same themes/categories allowing them to be presented together.

The narrative produced was guided by the Best Evidence Medical Education (BEME) Collaboration guide [64, 65] and the STORIES (STructured apprOach to the Reporting In healthcare education of Evidence Synthesis) statement [66]. Critical appraisals were deemed not necessary for this scoping review as it aimed to consider a wide landscape of articles and thus did not seek to exclude articles through critical appraisal scores.

### Stage 6: consultation with key stakeholders

Feedback was sought from key stakeholders after the results were collated and reported.

## Results

**Fig. 2:**
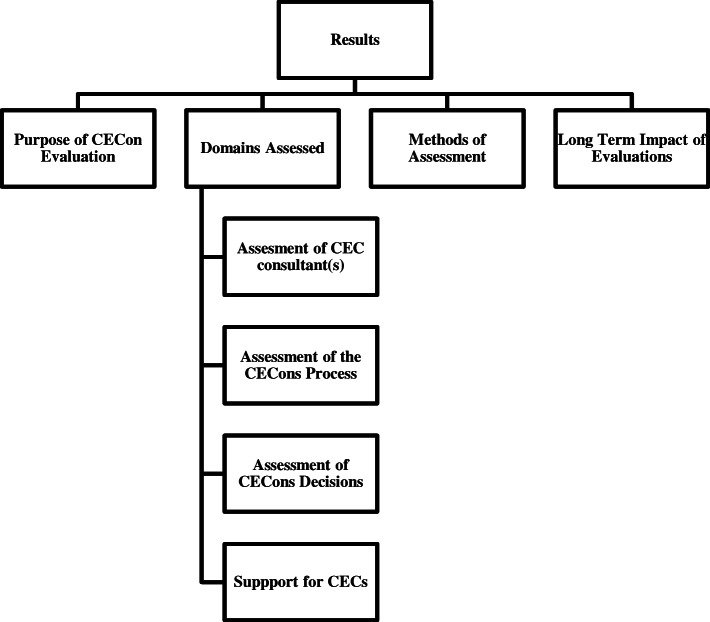
Results of This Review

The four themes/categories elucidated were the purpose of the CECons evaluation, the various domains assessed, the methods of assessment used and the long-term impact of these evaluations. These are outlined in Fig. 2*.*

1. ***Purpose of CECon evaluation***

The primary reason for evaluating CECons was to certify [67–69] or accredit [9] CEC consultants to ensure that they possessed the ‘necessary competencies’ [70]. CECons were also assessed to determine their impact on patient care [18, 71–75] and benchmark their programs against prevailing standards [17, 76, 77].

2. ***Domains assessed***

Four domains were assessed: the consultant(s), consultation process, decisions made, and support provided.

i. *Assessment of CEC consultant(s)*

The CEC consultant’s character, performance and values were seen to influence the perception, process and outcome of CECons. Desired personal attributes included tolerance, patience, compassion, honesty, self-knowledge, courage, prudence, humility, and integrity [70, 78–80]. Professional proficiencies sought after included prior training and experience with CEC practices [7, 16, 79, 81–86], ethico-legal knowledge [69, 78–81, 83, 86–96] and active involvement in promoting awareness of ethical issues in the clinical environment [80, 91, 92]. An effective CEC consultant also showed interest in organizational ethics [7], educating and guiding others on ethical issues [80, 91, 92] and aligning expectations and practice with institutional and CEC standards [82, 86]. Some studies evaluated all members of the CECons as a whole [22, 33, 97] and their ability to provide a broad review of ethico-legal and moral considerations [73, 98].

ii. *Assessment of the CECons process*

The CECons process was assessed through the approaches they employed such as whether all relevant information had been gathered [5, 14, 99–103] and that all ethical [5, 14, 73, 79, 90, 99–106] and moral issues [100, 102] were considered and analyzed together holistically [100, 102] in an objective [103] and accountable manner [107] whilst maintaining confidentiality [15, 79, 90, 103, 108].

Considerations also included if they were timely [31, 79, 109, 110], structured and accessible to all stakeholders [5, 9, 31]. In addition to conflicting or alternative positions taken up during the CECons [5, 14, 99–103], details of the deliberation process and whether consensus was reached on guidance provided were also assessed [100]. The presence of clear communication between consultants, healthcare professionals, patients and their families [5, 77, 79, 91, 92, 99, 111–117], the manner in which medical information was interpreted for these stakeholders [79, 111, 112] and if the ‘*most important information* [5]*’* was effectively articulated to them were also assessed.

Stakeholder satisfaction [9, 30, 85, 91, 116], their perceived helpfulness [74, 75, 89, 91, 105, 109, 114–118] and timeliness [15, 88, 98, 116, 119] were likewise considered. The levels of stress they experienced during CECons participation [74, 75, 91, 119] and the likelihood that users would recommend the CECs service to others [15, 75, 85, 89, 91, 109, 112, 116, 120] also served as a marker of their satisfaction.

iii. *Assessment of CECons decisions*

CECons decisions were assessed on whether

- they were perceived to be effective overall [5, 7, 111, 112, 114]
- they considered healthcare cost, clinical outcomes and treatment decisions in guidance provided [14, 17, 18, 73–75, 91, 121–125]
- guidance enhanced the healthcare professionals’ ethical competency [31, 71, 72, 91, 118, 123, 124]
- guidance adhered to organizational standards [71] and regarded as ethically, contextually, socioculturally and practically appropriate [5, 14, 73, 88, 99–101, 105]
- they were communicated to various stakeholders involved [5, 31]
- they were easy to understand by stakeholders, accessible to stakeholders and structured [5, 9]
- they successfully influenced patient care provided [7, 74, 75, 92, 99, 105, 108, 112, 114, 116, 117, 119–121] through assessment and recommendation of improvements [7, 30, 75, 105, 108]

iv. *Support for CECs*

The provision of financial and administrative support [108, 126, 127] for the CEC was often assessed and seen to impact its ability to provide effective consultations [88, 103, 108, 126, 127].

3. ***Methods of Assessment***

Assessments were carried out through:

i. Self-appraisals by CEC consultants [79, 82]
ii. Appraisals by external CEC members not involved in the particular CECons [76, 99, 108, 128, 129]
iii. Feedback from patients and family members [14, 69, 74, 91, 92, 114, 130, 131]
iv. Input from healthcare professionals [5, 14, 15, 33, 72, 74, 80, 85, 87, 90, 91, 98, 103, 111, 112, 114, 118, 119, 127, 130]
v. Evaluations by senior clinicians [82, 84, 93, 102, 104, 127]
vi. Evaluations by administrators or organizational representatives [77]

Assessments methods also include:

i. Longitudinal assessments that take place over many years [72]
ii. Single time point assessments
  - focus group interviews [14, 74, 76, 77, 84, 87, 90, 92, 98, 103, 127, 130]
  - questionnaires [33, 72, 74, 79, 85, 91, 92, 102, 111, 112, 114, 119, 127]
iii. Specific appraisal of CECons decisions

- case report analysis [5, 15, 77, 93, 100, 101, 104, 106, 108, 114, 122, 128, 129, 132]
- impact of CECons decisions such as via randomized controlled trials where an intervention group who received CECons were compared to a control group who did not [30, 74, 75, 99, 121, 122]

iv. Assessment of CECons’ shortcomings [85, 98, 103, 107, 126]
v. Documentation of CEC training and experience by consultants through portfolios and their subsequent review by senior ethics consultants and faculty [16, 82, 86]
  Number of referrals made to CEC services, viewed as an endorsement of their effectiveness and reputation [89].

4. ***Long-term Impact of CECons Evaluations***

Positive long-term impact of CECons evaluations include the development of new guidelines [15], formalization of ethics consultations [69], and increased self-reflection by CEC consultants [76, 108].

### Agreement of results by key stakeholders

The stakeholders and expert team were in agreement that these findings reflected prevailing practice and called for the design of a new holistic and longitudinal assessment tool for CECons based upon the disparate findings of this review.

## Discussion

In addressing its primary and secondary research questions, this systematic scoping review identifies a variety of tools designed to assess different aspects of CECons. The diversity of these assessment tools stem from the overall goals of assessing CECons which are largely driven by a combination of objectives including accrediting CEC members, evaluating the CECons process and benchmarking it against prevailing standards and/or other programs, and determining their overall outcomes on patient care.

Notably, the four domains assessed were the CEC personnel’s attributes and skillsets; the approach employed in the CECons process; the CECons decisions; and the presence of support for the CECs. This explains focus upon.

- the personal and professional attributes of CEC members [7, 16, 79, 81–86] and the composition, training, experience [70, 78–80] and skillset of the team [69, 78–81, 83, 86–96] carrying out the CECons (92–94);
- the approach adopted and if it considered the ethical, legal, moral, financial, clinical and professional issues holistically and objectively, and whether the process considered prevailing sociocultural and practical issues [5, 14, 73, 88, 99–101, 105] in a confidential manner [15, 79, 90, 103, 108]. Also considered was if the CECons was timely [31, 79, 109, 110], well-documented, structured, accessible [5, 9, 31], clearly communicated [5, 77, 79, 91, 92, 99, 111–117] to stakeholders and perceived as satisfactory through recommendations to others [15, 75, 85, 89, 91, 109, 112, 116, 120];
- the CECons decisions and if stakeholders found them effective [74, 75, 89, 91, 105, 109, 114–118], situationally appropriate [5, 14, 73, 88, 99–101, 105] and well-communicated [5, 31], if the guidance provided was educational and enhanced their ethical competency [31, 71, 72, 91, 118, 123, 124] and if it improved patient outcomes [7, 30, 75, 105, 108] and had long-term effects on their practice [76, 108];
- and the adequate provision of financial and administrative support [108, 126, 127] deemed to bolster the program and impact the CECs capacity to provide effective consultations [88, 103, 108, 126, 127].

With tools ranging from self-appraisals to single time point and longitudinal assessments, perhaps just as significant is the diversity in methods and the quality and type of data generated from them. Such variability in these domains and tools used explains the lack of consistency in CECons assessments. Whilst it may be argued that such diversity merely reflect practical limitations [133] or adaptations to sociocultural and clinical factors [134], a minimum standard must be upheld to ensure baseline efficacy. CEC programs must be rigorously structured and core competencies for CEC members consistently adopted. The *Healthcare Ethics Consultant-Certified Program* (HEC-C) and *Core Competencies for Healthcare Ethics Consultation* set out by the ASBH would set the tone for such structuring and training [135] and establish minimum data sets to be evaluated as well as guide standardization of methods used to collect the data [128].

However such a standard setting process must be mindful of the prevailing clinical, educational, ethical, legal, sociocultural, financial and contextual factors influencing the CECs as they continue to expand across North America [6, 7], Asia [8–12] and Europe [13–15]. It may prove useful for CECs to adopt a Modified Delphi approach to consider the key elements of an effective CECons process and design an assessment tool that better suits their context, focus, capabilities and capacities [118].Indeed, like pieces of a jigsaw, bringing together and carefully deliberating the disparate considerations and domains discerned by this systematic scoping review would allow for a more cohesive and comprehensive assessment tool to be curated.

### Limitations

There are a number of limitations to this review. Firstly, use of the directed content analysis based on a relatively unique interpretation of the data would have been problematic without the employment of the ‘Split Approach’. However, whilst the ‘Split Approach’ addresses concerns surrounding the validity of using a directed content analysis and addresses researcher reflexivity, this approach remains unproven. Despite this, some of these concerns are assuaged with the use of Braun and Clarke’s (2006) [61] approach to thematic analysis, which served as a means of confirming the evidence, a form of triangulation and a method of enhancing the validity of the findings.

Secondly, this review drew conclusions from a small pool of papers which were limited to articles published or translated into the English language, primarily from North America and Europe. This may limit the applicability of the findings in wider healthcare settings.

## Conclusion

In addressing its primary and secondary research questions, this systematic scoping review highlights the variable goals, contextual factors and practical limitations behind the lack of a consistent approach to assessing CECons. In so doing this review also highlights the need for the design of a contextually appropriate, culturally sensitive, program specific assessment tool designed around the key domains identified here to be used not only to evaluate the quality and content of CECons but as a means of informing the training and assessment of CEC members, improving CECons procedures, assessing the efficacy and impact of its CECons and benchmarking its practice with other programs and international standards of practice.

With enhancing patient care at the centre of its processes, CECs should employ prevailing design principles and assessment theories to improve its educational and policy making roles in establishing national standards. Whilst there is still much to be done, and the efficacy of CEC’s other roles to be evaluated, we believe this systematic scoping review points the way towards more accountable, effective and user-friendly CECs. We look forward to further discourse on this critical aspect of clinical practice.

## Supplementary information

**Additional file 1.** Appendix with PubMed Search Strategy and List of Included Articles.

## Data Availability

All data generated or analysed during this study are included in this published article.

## Abbreviations

CECs: Clinical Ethics Committees
CECons: Clinical Ethics Consultations
