# Supplementary material for: Evaluating assessment tools of the quality of clinical ethics consultations: a systematic scoping review from 1992 to 2019
Source: BMC Med Ethics. 2020 Jul 1;21:51. doi: 10.1186/s12910-020-00492-4 (PMC7329412; doi:10.1186/s12910-020-00492-4)
Supplement: Supplementary file 1 — Additional file 1. Appendix with PubMed Search Strategy and List of Included Articles. [file 12910_2020_492_MOESM1_ESM.docx]

**Appendix**

Table 1: Detailed PubMed Search Strategy

| PubMed | ( ( (“Ethics, Clinical"[Mesh] OR "Clinical Decision-Making/ethics"[Mesh]  OR "Decision Making/ethics"[Mesh] OR "Evidence-Based Medicine/ethics"[Mesh] OR Physicians/ethics “Ethics, Medical"[Mesh] OR “Ethics, Clinical"[Mesh] OR "Clinical Decision-Making/ethics"[Mesh])  AND  ("Ethics Consultation"[Mesh] OR "Ethical Review"[Mesh] OR "Ethics Committees"[Mesh] OR “Ethicists"[Mesh] OR "Referral and Consultation/ethics"[Mesh])  )  OR    (  (clinical ethic*[tiab] OR medical ethic*[tiab] OR medicine ethic*[tiab])  AND  (consult*[tiab] OR Committee*[tiab] OR review*[tiab]) )   OR   (  (ethics consult*[tiab] OR ethical consult*[tiab])  AND  (clinical[tiab] OR medical[tiab] OR medicine[tiab])  ) ) |
| --- | --- |

Table 2: List of Included Articles

| No. | Title of Article | Authors | Year |
| --- | --- | --- | --- |
| 1 | The question of method in ethics consultation | Agich, George J | 2001 |
| 2 | Development of a Clinical Ethics Committee De Novo at a Small Community Hospital by Addressing Needs and Potential Barriers | Arzuaga, Bonnie H | 2017 |
| 3 | Outcomes of ethics consultations in adult ICUs: a systematic review and meta-analysis | Au, Selena S  Couillard, Philippe  Roze des Ordons, Amanda  Fiest, Kirsten M  Lorenzetti, Dianne L  Jette, Nathalie | 2018 |
| 4 | Health care ethics consultation: Nature, goals, and competencies: A position paper from the society for health and human values–society for bioethics consultation task force on standards for bioethics consultation | Aulisio, Mark P  Arnold, Robert M  Youngner, Stuart J | 2000 |
| 5 | Discussing end-of-life decisions in a clinical ethics committee: an interview study of Norwegian doctors’ experience | Bahus, Marianne K  Førde, Reidun | 2016 |
| 6 | Lessons learned from nurses’ requests for ethics consultation: Why did they call and what did they value? | Bartlett, Virginia L  Finder, Stuart G | 2018 |
| 7 | Assessing clinical ethics consultation: processes and outcomes | Batten, Jason | 2013 |
| 8 | The Next Step for Quality Attestation | Bayley, Carol | 2013 |
| 9 | Ethics committee networks: Facing the future | Beltran, Joseph E | 1995 |
| 10 | Searching for effectiveness: the functioning of Connecticut Clinical Ethics Committees | Berchelmann, Kathleen  Blechner, Barbara | 2002 |
| 11 | Quality assessment of the ethics consultation service at the organizational level: accrediting ethics consultation services | Berkowitz, Kenneth A  Katz, Aviva L  Powderly, Kathleen E  Spike, Jeffrey P | 2016 |
| 12 | Not There Yet: Evaluating Clinical Ethics Consultation in an Accountability Culture | Bruce, Courtenay R  Bibler, Trevor M | 2016 |
| 13 | “Systematizing” ethics consultation services | Bruce, Courtenay R  Eves, Margot M  Allen, Nathan G  Smith, Martin L  Peña, Adam M  Cheney, John R  Majumder, Mary A | 2015 |
| 14 | An embedded model for ethics consultation: Characteristics, outcomes, and challenges | Bruce, Courtenay R  Peña, Adam  Kusin, Betsy B  Allen, Nathan G  Smith, Martin L  Majumder, Mary A | 2014 |
| 15 | Towards Substantive Standardization: Ethical Rules as Ethical Presumptions | Chan, Benjamin | 2016 |
| 16 | Evaluating ethics consultation: randomised controlled trial is not the right tool | Chen, Yen-Yuan  Chen, Yen-Chang | 2008 |
| 17 | To evaluate the effectiveness of health care ethics consultation based on the goals of health care ethics consultation: a prospective cohort study with randomization | Chen, Yen-Yuan  Chu, Tzong-Shinn  Kao, Yu-Hui  Tsai, Pi-Ru  Huang, Tien-Shang  Ko, Wen-Je | 2014 |
| 18 | Should ethics committees study themselves? | Danis, Marion | 1994 |
| 19 | ‘I just love these sessions’. Should physician satisfaction matter in clinical ethics consultations? | Delany, Clare  Hall, Georgina | 2012 |
| 20 | The art of the chart note in clinical ethics consultation and bioethics mediation: conveying information that can be understood and evaluated | Dubler, Nancy Neveloff | 2013 |
| 21 | Charting the future: Credentialing, privileging, quality, and evaluation in clinical ethics consultation | Dubler, Nancy Neveloff  Webber, Mayris P  Swiderski, Deborah M  Faculty  the National Working Group for the Clinical Ethics Credentialing Project | 2009 |
| 22 | A national survey of US internists’ experiences with ethical dilemmas and ethics consultation | DuVal, Gordon  Clarridge, Brian  Gensler, Gary  Danis, Marion | 2004 |
| 23 | The main indicators for Iranian hospital ethical accreditation | Enjoo, Seyed Ali  Amini, Mitra  Tabei, Seyed Ziaadin  Mahbudi, Ali  Kavosi, Zahra  Saber, Mahboobeh | 2015 |
| 24 | The “Quality Attestation” Process and the Risk of the False Positive | Fiester, Autumn | 2014 |
| 25 | Discovering what matters: interrogating clinician responses to ethics consultation | Finder, Stuart G  Bartlett, Virginia L | 2017 |
| 26 | A pilot evaluation of portfolios for quality attestation of clinical ethics consultants | Fins, Joseph J  Kodish, Eric  Cohn, Felicia  Danis, Marion  Derse, Arthur R  Dubler, Nancy Neveloff  Goulden, Barbara  Kuczewski, Mark  Mercer, Mary Beth  Pearlman, Robert A | 2016 |
| 27 | Developing and testing a checklist to enhance quality in ethics consultation | Flicker, Lauren Sydney  Rose, Susannah L  Eves, Margot M  Flamm, Anne Lederman  Sanghani, Ruchi  Smith, Martin L | 2014 |
| 28 | Evaluation of case consultations in clinical ethics committees | Førde, Reidun  Pedersen, Reidar | 2012 |
| 29 | Clinicians’ evaluation of clinical ethics consultations in Norway: a qualitative study | Førde, Reidun  Pedersen, Reidar  Akre, Victoria | 2008 |
| 30 | The Road to Certification for Clinical Ethics Consultants: Finding Our Bearings | Fox, Ellen | 2016 |
| 31 | Residents’ access to ethics consultations: knowledge, use, and perceptions | Gacki-Smith, Jessica  Gordon, Elisa J | 2005 |
| 32 | The development of a clinical policy ethics assessment tool | Garcia-Capilla, Diego José  Rubio-Navarro, Alfonso  Torralba-Madrid, Maria José  Rutty, Jane | 2019 |
| 33 | Barriers and facilitators to consulting hospital clinical ethics committees | Gaudine, Alice  Lamb, Marianne  LeFort, Sandra M  Thorne, Linda | 2011 |
| 34 | Evolution of hospital clinical ethics committees in Canada | Gaudine, Alice  Thorne, Linda  LeFort, Sandra M  Lamb, Marianne | 2010 |
| 35 | The CASES Approach to Ethics Consultation: The Centrality of the Ethics Question | Geppert, Cynthia  Chanko, Barbara L | 2016 |
| 36 | What attributes should clinical ethics committees have? | Gillon, Raanan | 2010 |
| 37 | A better way to evaluate clinical ethics consultations? An ecological approach | Gordon, Elisa J | 2007 |
| 38 | Hospital ethics committees: problems in evaluation | Griener, Glenn G  Storch, Janet L | 1992 |
| 39 | Evaluating clinical ethics support in mental healthcare: a systematic literature review | Hem, Marit Helene  Pedersen, Reidar  Norvoll, Reidun  Molewijk, Bert | 2015 |
| 40 | Evaluation of a paediatric clinical ethics service | Jansen, Melanie A  Schlapbach, Luregn J  Irving, Helen | 2018 |
| 41 | Physicians' attitudes toward clinical ethics consultation: a research study from Turkey | Kadioğlu, Funda Gülay  Can, Rana  Okuyaz, Selda  Yalçin, Sibel Öner  Kadioğlu, Nuri Selim | 2011 |
| 42 | Quality attestation for clinical ethics consultants: A two‐step model from the American Society for Bioethics and Humanities | Kodish, Eric  Fins, Joseph J  Braddock III, Clarence  Cohn, Felicia  Dubler, Nancy Neveloff  Danis, Marion  Derse, Arthur R  Pearlman, Robert A  Smith, Martin  Tarzian, Anita | 2013 |
| 43 | Community hospital ethics consultation: evaluation and comparison with a university hospital service | La Puma, John  Stocking, Carol B  Darling, Cheryl M  Siegler, Mark | 1992 |
| 44 | Quality control for hospitals' clinical ethics services: proposed standards | Leeman, Cavin P  Fletcher, John C  Spencer, Edward M  Fry-Revere, Sigrid | 1997 |
| 45 | Networking healthcare ethics committees: Benefits and obstacles | Loeben, Greg S | 1999 |
| 46 | Quality in ethics consultations | Magill, Gerard | 2013 |
| 47 | Evaluation of a medical ethics consultation service: opinions of patients and health care providers | McClung, John A  Kamer, Russell S  DeLuca, Margaret  Barber, Harlan J | 1996 |
| 48 | Evaluating clinical ethics support: a participatory approach | Metselaar, Suzanne  Widdershoven, Guy  Porz, Rouven  Molewijk, Bert | 2017 |
| 49 | Is evaluating ethics consultation on the basis of cost a good idea? | Mills, Ann E  Tereskerz, Patricia  Davis, Walt | 2005 |
| 50 | Pediatricians' experience with clinical ethics consultation: a national survey | Morrison, Wynne  Womer, James  Nathanson, Pamela  Kersun, Leslie  Hester, D Micah  Walsh, Corbett  Feudtner, Chris | 2015 |
| 51 | Ethics program evaluation: The Virginia hospital ethics fellows example | Neff-Smith, Martha  Giles, Scott  Spencer, Edward M  Fletcher, John C | 1997 |
| 52 | Reinvigorating ethics consultations: an impetus from the “quality” debate | Nilson, Elizabeth G Fins, Joseph J | 2006 |
| 53 | Effectiveness of an ethics consultation service | Orr, Robert D  Moon, Eliot | 1993 |
| 54 | Evaluation of an ethics consultation service: patient and family perspective | Orr, Robert D  Morton, Kelly R  deLeon, Dennis M  Fals, Juan C | 1996 |
| 55 | Ethics consultation quality assessment tool: A novel method for assessing the quality of ethics case consultations based on written records | Pearlman, Robert A  Foglia, Mary Beth  Fox, Ellen  Cohen, Jennifer H  Chanko, Barbara L  Berkowitz, Kenneth A | 2016 |
| 56 | Barriers and challenges in clinical ethics consultations: the experiences of nine clinical ethics committees | Pedersen, Reidar  Akre, Victoria  Førde, Reidun | 2009 |
| 57 | The development of a descriptive evaluation tool for clinical ethics case consultations | Pedersen, Reidar  Hurst, SA  Schildmann, Jan  Schuster, Sandra  Molewijk, Bert | 2010 |
| 58 | Evaluating clinical ethics consultation: a European perspective | Pfäfflin, Margarete  Kobert, Klaus  Reiter-Theil, Stella | 2009 |
| 59 | Does quality attestation come in only one size? | Postema, Don C | 2013 |
| 60 | Meaningful Use of Electronic Health Records for Quality Assessment and Review of Clinical Ethics Consultation | Sanelli-Russo, Susan  Folkers, KM  Sakolsky, William  Fins, Joseph J  Dubler, Nancy Neveloff | 2018 |
| 61 | Evaluation of clinical ethics support services and its normativity | Schildmann, Jan  Molewijk, Bert  Benaroyo, Lazare  Forde, Reidun  Neitzke, Gerald | 2013 |
| 62 | Impact of ethics consultations in the intensive care setting: a randomized, controlled trial | Schneiderman, Lawrence J  Gilmer, Todd  Teetzel, Holly D | 2000 |
| 63 | Effect of ethics consultations on nonbeneficial life-sustaining treatments in the intensive care setting: a randomized controlled trial | Schneiderman, Lawrence J  Gilmer, Todd  Teetzel, Holly D  Dugan, Daniel O  Blustein, Jeffrey  Cranford, Ronald  Briggs, Kathleen B  Komatsu, Glen I  Goodman-Crews, Paula  Cohn, Felicia | 2003 |
| 64 | Dissatisfaction with ethics consultations: The Anna Karenina principle | Schneiderman, Lawrence J  Gilmer, Todd  Teetzel, Holly D  Dugan, Daniel O  Goodman-Crews, Paula  Cohn, Felicia | 2006 |
| 65 | The application of standards and recommendations to clinical ethics consultation in practice: an evaluation at German hospitals | Schochow, Maximilian  Rubeis, Giovanni  Steger, Florian | 2017 |
| 66 | Clinical ethics support services in the UK: an investigation of the current provision of ethics support to health professionals in the UK | Slowther, Anne  Bunch, Chris  Woolnough, Brian  Hope, Tony | 2001 |
| 67 | Criteria for determining the appropriate method for an ethics consultation | Smith, Martin L  Bisanz, Annette K  Kempfer, Ana J  Adams, Barbie  Candelari, Toya G  Blackburn, Roxann K | 2004 |
| 68 | Quality of ethical guidelines and ethical content in clinical guidelines: the example of end-of-life decision-making | Strech, Daniel  Schildmann, Jan | 2011 |
| 69 | Five-year experience of clinical ethics consultations in a pediatric teaching hospital | Streuli, Jürg C  Staubli, Georg  Pfändler-Poletti, Marlis  Baumann-Hölzle, Ruth  Ersch, Jörg | 2014 |
| 70 | Health care ethics consultation competences and standards: a roadmap still needing a compass | Swetz, Keith M  Hook, C Christopher  Henriksen Hellyer, Joan M  Mueller, Paul S | 2013 |
| 71 | The clinical ethics credentialing project: Preliminary notes from a pilot project to establish quality measures for ethics consultation | Swiderski, Deborah M  Ettinger, Katharine M  Webber, Mayris  Dubler, Nancy N | 2010 |
| 72 | Evaluating ethics consultation: framing the questions | Tulsky, James A  Fox, Ellen | 1996 |
| 73 | Obstacles and opportunities in the design of ethics consultation evaluation | Tulsky, James A  Stocking, Carol B | 1996 |
| 74 | Ongoing Evaluation of Clinical Ethics Consultations as a Form of Continuous Quality Improvement | Volpe, Rebecca | 2017 |
| 75 | Methodological reflections on the contribution of qualitative research to the evaluation of clinical ethics support services | Wäscher, Sebastian  Salloch, Sabine  Ritter, Peter  Vollmann, Jochen  Schildmann, Jan | 2017 |
| 76 | Moral Reasoning among HEC Members: An Empirical Evaluation of the Relationship of Theory and Practice in Clinical Ethics Consultation | Wasserman, Jason Adam  Stevenson, Shannon Lindsey  Claxton, Cassandra | 2015 |
| 77 | Developing an evaluation tool for assessing clinical ethics consultation skills in simulation based education: The ACES project | Wasson, Katherine  Parsi, Kayhan  McCarthy, Michael  Siddall, Viva Jo  Kuczewski, Mark | 2016 |
| 78 | Structuring a written examination to assess ASBH health care ethics consultation core knowledge competencies | White, Bruce D  Jankowski, Jane B  Shelton, Wayne N | 2014 |
| 79 | A practical instrument to evaluate ethics consultations | White, Jocelyn C  Dunn, Patrick M  Homer, Lou | 1997 |
| 80 | Empirical assessments of clinical ethics services: implications for clinical ethics committees | Williamson, Laura | 2007 |
| 81 | Outcomes of ethics consultations in adult ICUs: A systematic review and meta-analysis | Au, S. S.  Couillard, P.  Roze Des Ordons, A.  Fiest, K. M.  Lorenzetti, D. L.  Jette, N. | 2018 |
| 82 | Nurses performance in clinical ethics committees and commissions: An integrative review | de Brito, G. M. G.  de Oliveira Santa Rosa, D. | 2019 |
| 83 | Defining and categorizing outcomes of Moral Case Deliberation (MCD): concept mapping with experienced MCD participants | de Snoo-Trimp, J. C.  Molewijk, B.  de Vet, H. C. W. | 2018 |
| 84 | Field-testing the Euro-MCD Instrument: Experienced outcomes of moral case deliberation | de Snoo-Trimp, J. C.  Molewijk, B.  Ursin, G.  Brinchmann, B. S.  Widdershoven, G. A.  de Vet, H. C.  Svantesson, M. | 2019 |
| 85 | Falling on deaf ears: a qualitative study on clinical ethical committees in France | Dekeuwer, C.  Bogaert, B.  Eggert, N.  Harpet, C.  Romero, M. | 2019 |
| 86 | Clinical ethics consultations in psychiatric compared to non-psychiatric medical settings: characteristics and outcomes | Lobbing, T.  Carvalho Fernando, S.  Driessen, M.  Schulz, M.  Behrens, J.  Kobert, K. K. B. | 2019 |
| 87 | Certification and evaluation of the clinical ethics consultant. A proposal for Italy | Picozzi, M.  Gasparetto, A.  Nicoli, F.  Pegoraro, R. | 2018 |
| 88 | Evaluating Clinical Ethics Support: On What Grounds Do We Make Judgments About Reports of Ethics Consultation? | Reiter-Theil, S.  Schurmann, J. | 2018 |
| 89 | Meaningful Use of Electronic Health Records for Quality Assessment and Review of Clinical Ethics Consultation | Sanelli-Russo, S.  Folkers, K. M.  Sakolsky, W.  Fins, J. J.  Dubler, N. N. | 2018 |
| 90 | Team members perspectives on conflicts in clinical ethics committees | Scherer, A.  Alt-Epping, B.  Nauck, F.  Marx, G. | 2019 |
| 91 | Do we understand the intervention? What complex intervention research can teach us for the evaluation of clinical ethics support services (CESS) | Schildmann, J.  Nadolny, S.  Haltaufderheide, J.  Gysels, M.  Vollmann, J.  Bausewein, C. | 2019 |
| 92 | The ASBH Approach to Certify Clinical Ethics Consultants Is Both Premature and Inadequate | Siegler, M. | 2019 |
| 93 | Quality standards for clinical ethics consultation | Simon, A. | 2009 |
| 94 | Certification ISO 9001 in clinical ethics consultation for improving quality and safety in healthcare | Tozzo, P.  Mazzi, A.  Aprile, A.  Rodriguez, D.  Caenazzo, L. | 2018 |
| 95 | Lessons learned from implementing a responsive quality assessment of clinical ethics support | van Baarle, E. M.  Potma, M. C.  van Hoek, M. E. C.  Hartman, L. A.  Molewijk, B. A. C.  van Gurp, J. L. P. | 2019 |
| 96 | Peer Review and Beyond: Towards a Dialogical Approach of Quality in Ethics Support | Widdershoven, G. A. M.  Molewijk, B.  Metselaar, S. | 2018 |
| 97 | Impact of pediatric ethics consultations on patients, families, social workers, and physicians | Yen, Bertina M  Schneiderman, Lawrence J | 1999 |
| 98 | Importance of systematic deliberation and  stakeholder presence: a national study of clinical  ethics committees | Morten Magelssen,  Reidar Pedersen, Ingrid Miljeteig, Håvard Ervik,  Reidun Førde | 2019 |
| 99 | Hospital/clinical ethics committees' notion: an overview | Fatemeh Hajibabaee, Soodabeh Joolaee,  Mohammad Ali Cheraghi,  Pooneh Salari,  Patricia Rodney | 2016 |
| 100 | The performance of the ethics committees in teaching hospitals affiliated with Mashhad University of Medical Sciences. | Meraji M,  Sadoughi F,  Ramezan Ghorbani N,  Nezami A. | 2014 |
| 101 | “It scares me to know that we might not have been there!”: a qualitative study into the experiences of parents of seriously ill children participating in ethical case discussions. | Førde R,  Linja T. | 2015 |
| 102 | Ethical case interventions for adult patients. | Schildmann J, Nadolny S, Haltaufderheide J et al. | 2019 |
| 103 | Outcomes of clinical ethics support near the end of life: A systematic review. | Haltaufderheide J, Nadolny S, Gysels M et al. | 2019 |
| 104 | Outcomes of moral case deliberation--the development of an evaluation instrument for clinical ethics support (the Euro-MCD). | Svantesson M, Karlsson J, Boitte P et al. | 2014 |
